# Idiosyncratic reaction after injection of polyacrylate - polyalcohol copolymer

**DOI:** 10.1590/S1677-5538.IBJU.2017.0446

**Published:** 2018

**Authors:** Cristiano Linck Pazeto, Fábio José Nascimento, Lucila Heloisa Simardi Santiago, Sidney Glina

**Affiliations:** 1 Departamento de Urologia, Faculdade de Medicina do ABC (FMABC), Santo André, SP, Brasil; 2 Departamento de Patologia, Faculdade de Medicina do ABC (FMABC), Santo André, SP, Brasil

**Keywords:** lidocaine-polyacrylate [Supplementary Concept], Vesico-Ureteral Reflux, Hyperplasia

## Abstract

**Context:**

Polyacrylate-polyalcohol copolymer is a synthetic product, non-biodegradable, with low rate of therapeutic failure and lower incidence of reactions at the site of injection, when compared to biodegradable agents. We report an unprecedent, exuberant and persistent inflammatory reaction following injection of that substance.

**Patient:**

a 17 years-old patient with vesico-ureteral reflux and complete pyelocaliceal right duplication was submitted to treatment with polyacrylate-polyalcohol copolymer (STING technique). In the seventh day of post-operatory, she presented intense dysuria and hypogastric pain, without laboratory exams alterations; a symptomatic treatment was started. After two months, the symptoms persisted and an ultrasound detected thickening of bladder wall close to the uretero-vesical junction. After that exam, a cystostopic biopsy showed epithelial hyperplasia with increased edema of lamina propria, suggesting an adverse reaction to the polymer. After four months, there was complete remission, but the reflux persisted with the same grade.

**Hypothesis:**

This is an unprecedent reaction following injection of this copolymer. The presence of characteristics such as absence of infection, temporal relation between treatment and beginning of symptoms, and detection of epithelial hyperplasia at the local of injection reinforce the hypothesis of association of the substance and adverse reaction. In that patient, important complains motivated early investigation of urinary tract, that confirmed those aspects. Maybe if that reaction had occurred in patients with lower capacity of expression (such as in infants) it would be unnoticed.

## SCENARIO

**Figure 1:**
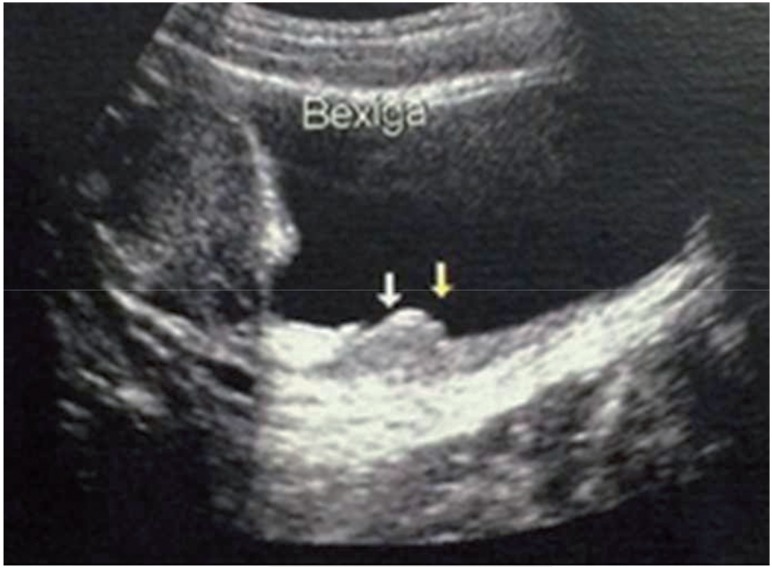
Focal thickening of 3.0 × 3.0cm in the bladder wall at the level of the right uretero-vesical junction.

**Figure 2:**
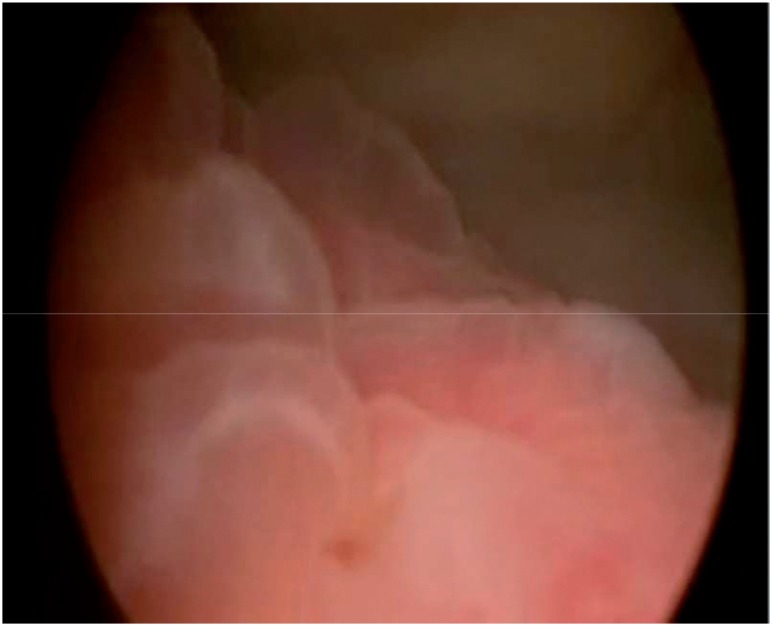
High hyperemic, bullous lesion with size and location similar to those described on ultrasonography.

**Figure 3:**
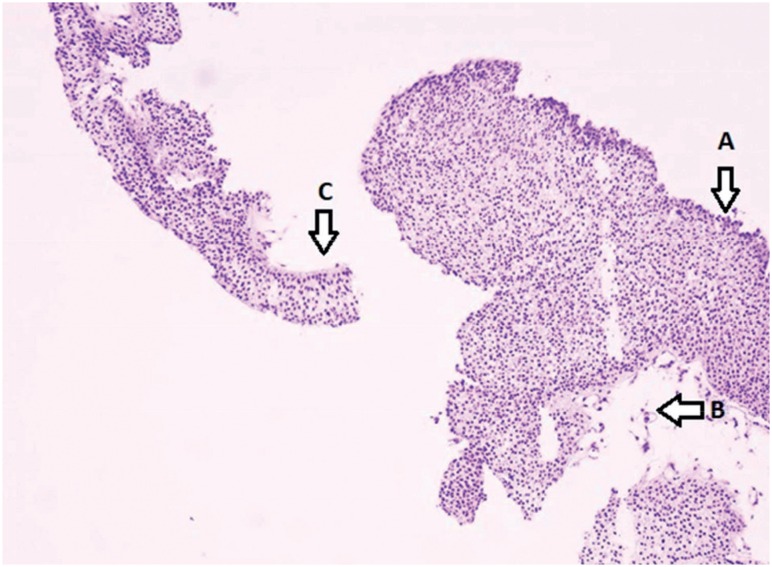
Epithelial hyperplasia (Arrow A), marked blade edema (Arrow B). compare with normal left fragment (Arrow c). HE 100X.

**Figure 4:**
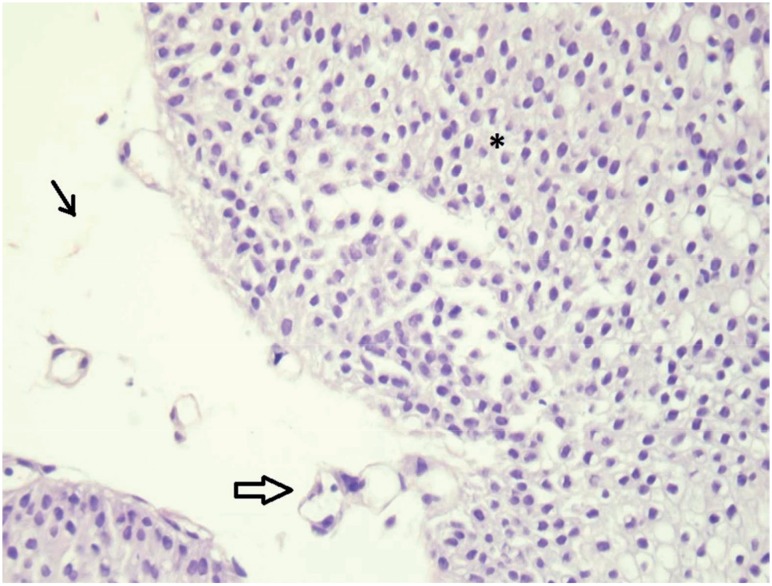
Observe hyperplastic urothelium with several layers of cells (*). Blade itself with marked edema (fine arrow) and dilated capillaries (thick arrow). HE 400X.

A female 17 years-old patient presented for urologic consultation with history of repeated cystitis and acute pyelonephritis (in the last episode it was necessary intensive care). She denied micturition and intestinal complaints as well as comorbidities. She referred that acute cystitis emerged after the beginning of sexual activity. Urethrocystogram showed the presence of vesicoureteral reflux grade II associated to complete right pyelocalicoureteral duplication (both ureters at that side showed reflux). DMSA-scintigraphy and blood and urinary exams were normal. After discussion of possible therapeutic interventions with the patient, it was opted for endoscopic treatment of reflux. During cystoscopy, it was identified two parallel ureteral meatus on the right side. Next, two wire-guides were introduced (one at each right ureteral meatus) in order to characterize the lower and superior units of the kidney. Then, using the STING technique (sub-ureteral injection), 1.5mL of polyacrylate-polyalcohol copolymer was injected at the lower meatus (single puncture obtaining correct volumetric effect). The detected increase following injection involved also the correspondent meatus of the superior unit. The procedure was carried out without any problems. However, after seven days of surgery, the patient presented with intense dysuria and hypogastric pain. In that moment, blood and urine exams were normal and it was started a symptomatic treatment. After two months, the complains persisted and an ultrasound showed focal thickening of 3.0×3.0cm at the bladder wall close to the right uretero-vesical junction (Figure-1). Due to this atypical and refractory presentation, it was performed a diagnostic cystoscopy, that showed an elevated lesion, hyperemic, of bullous aspect, with size and location similar to those described at ultrasound (Figure-2). The lesion was biopsied, and the pathologic exam showed epithelial hyperplasia with marked edema of lamina propria (Figures 3 and 4), suggesting a possible adverse reaction to polymer. For that reason, it was introduced betamethasone and anti-inflammatory drugs. After four months of the beginning of the symptoms, the patient completely improved. However, a new urethrocystogram showed persistence of same grade reflux.

## HYPOTHESIS

This is an unprecedent reaction of polyacrylate-polyalcohol copolymer injection. The intense inflammatory reaction at the site of injection could have been caused by any hypersensitivity to the substance. The factors that suggested that hypothesis include: absence of infection (several negative urine cultures), temporal relationship between treatment and beginning of symptoms, detection of epithelial hyperplasia coincident to the site of injection and significant improvement with the use of corticosteroid. Also, the thickening observed at ultrasound was much more intense than that usually observed at post-operatory. The significant complaints of the patients motivated early investigation of urinary tract, that detected those findings. Therefore, if the reaction was lighter or in patients with lower capacity of expression (such as in infants, for example), it could be unnoticed. Until the present, the patient does not present any other alterations aside from persistence of reflux during a six months follow-up.

## DISCUSSION

Polyacrylate-polyalcohol copolymer is a synthetic product of the acrylic family, nonbiodegradable, with high molecular weight, that, when injected, forms a fibrotic capsule-due to its stability and durability. These proprieties associated to its biocompatibility are the main advantages of that substance in relation to biodegradable agents. These last present a high rate of reabsorption, associated to higher rate of failure of treatment and allergic reactions (1).

Several studies evaluated the efficacy of polyacrylate-polyalcohol copolymer and showed high rates of reflux resolution, including more severe cases (2–4). After literature review, we did not identify any relationship between polyacrylate-polyalcohol copolymer and foreign body reaction or hypersensitivity. Inflammatory reaction observed in that patient could have been similar to rheumatologic diseases that affect the ureter (such as eosinophilic ureteritis), that could lead to ureteral obstruction, explaining some patients with late obstruction described with the use of that agent (5). Among known complications of the injection of that copolymer, it is observed ureteral obstruction (early or late), contralateral reflux and local calcification (6).

There are some evidences of granulomatous inflammatory reaction (typical of foreign body reaction) after injection of biodegradable agents. For example, in one study, some patients with persistence of reflux after injection of dextranomer/hyaluronic acid were submitted to ureteral reimplantation. During those procedures, it was collected samples of tissue of the region of the implant for histologic analysis, that showed high rate of eosinophilic infiltrate in 7 patients. These findings suggest the occurrence of hypersensitivity against some component of the used copolymer (7).

In relation to polyacrylate-polyalcohol copolymer, literature shows one 9 years-old patient with ureteral obstruction at post-operatory. In that case, during ureteral reimplantation, it was observed a lush inflammatory reaction (similar to a “tumor”), and the histologic exam described it as a pseudotumor inflammatory reaction with the presence of giant cells. It was suspected that the injection technique (HIT) and the high dose of polyacrylate (1.2mL) could have been the causes of that reaction (8).

Foreign body reaction, previously described after injection of dextranomer (7), is typically observed as a granulomatous inflammation with multinucleated giant cells and other inflammatory cells such as lymphocytes, mastocytes and some eosinophils. The reaction in this patient seems more lenient and there were not the alterations above.

Due to the presence of ureteral duplication and age of our patient, it was necessary to use a high volume of copolymer (1.5mL) to obtain a volumetric effect at the site of injection. That fact, along with the possible unprecedent reaction, could explain the adverse event. However, other authors have already used the same dose without this reaction (8, 9). Also, it was reported some complications such as obstruction, for example, with usual doses of polyacrylate-polyalcohol copolymer (0.5-1.0mL) and dextranomer/hyaluronic acid (0.7-1.2mL) (9, 10). In relation to the patient's age, although higher than most studied patients, there is no evidence that that fact may have collaborated for the event - in literature, there is one 32 years old patient that was injected, for example (9). Also, there is no reason to relate the reaction to therapeutic failure.

Complication in that patient was not accompanied of any sign of urinary obstruction, loss of renal function, or infection, and also, the patient responded well to conservative treatment without the need of other interventions. However, the important presented symptoms and the lack of data on the theme, difficulted the treatment. It is possible that such complication was an idiosyncratic reaction related to a specific susceptibility of that patient, instead of, for example, hypersensitivity. Anyway, this is an unprecedented case related to copolymer injection. At last, we highlight the importance of strict follow-up of those patients, in view of the great variety of early and late complications that not always present symptoms.

## ABBREVIATIONS

STING: = Subureteral transurethral injection
HIT: = Hydrodistension implantation technique
